# Chronic fungal sinusitis leading to disastrous cerebral aspergillosis: a case report

**DOI:** 10.1186/1757-1626-2-9406

**Published:** 2009-12-31

**Authors:** Muhammad J Popalzai, Anurag Kushawaha, Neville Mobarakai, Rohail Asrar, Farida Durrani

**Affiliations:** 1 Department of Medicine, Staten Island University Hospital, Division of Infectious Diseases 475 Seaview Avenue, Staten Island, NY 10305, USA

## Abstract

Cerebral angioinvasion is a fatal complication of disseminated aspergillosis and has been rarely described in diabetic population in the absence of ketoacidosis. We present a case of invasive fungal sinusitis in a 79 year old diabetic man who presented with chronic frontal headaches. Despite appropriate medical and surgical treatment, the disease progressed and the patient eventually succumbed to a fatal ruptured mycotic aneurysm. We emphasize that clinicians should consider this in the differential diagnoses of all diabetics who present with chronic sinusitis, as early diagnosis could be the key in the successful treatment.

## Introduction

Invasive cerebral aspergillosis is a rare manifestation of disseminated aspergillosis and is associated with high mortality rates. Ruptured mycotic aneurysm and subarachnoid hemorrhage are uncommon but devastating complications in invasive cerebral aspergillosis. The disease entity has been described in immunocompromised patients, but has only rarely been reported in diabetic patients. Chronic fungal sinusitis should be recognized as an early sign of possible aspergillosis and often presents with new-onset, persistent headaches. The ability of *Aspergillus *to attack blood vessels leading to necrotizing angiitis, secondary thrombosis, and hemorrhage, is a characteristic angioinvasive feature and thereby makes insidious aspergillosis an important consideration in persons manifesting with acute onset of focal neurologic deficits, including immunocompetent patients in the correct clinical setting. We present a fatal case report of a 79-year-old diabetic man who presented with relentless headaches who was found to have chronic sphenoid fungal sinusitis. Despite treatment, he eventually succumbed to a ruptured mycotic aneurysm that was the result of direct extension from chronic *Aspergillus *sinusitis to the intracerebral circulation.

## Case presentation

A 79-year-old man of Egyptian descent presented to the emergency department (ED) with complaints of chronic frontal headaches worsening over the last 2 weeks. The patient had a past medical history of hypertension, insulin-dependent diabetes mellitus, prostate cancer status-post prostatectomy, and osteoarthritis. He denied any history of steroid use or any chemotherapy. He denied recent travel. He had immigrated to the USA from Egypt eleven years before. He denied smoking, alcohol, or illicit drug use. He denied any allergies.

The history goes back one year prior when he began experiencing intermittent frontal headaches. Initially he underwent ophthalmologic evaluation which was normal. His symptoms progressed over the following months. He was evaluated several times in the ED during this time but workup was unrevealing. His headache had dramatically worsened 2 weeks ago accompanied by fever, malaise, nausea, vomiting and decreased appetite. He went to his primary medical doctor and was started on clarithromycin as an outpatient for the treatment of presumed otitis media. The symptoms did not improve after 5 days of antibiotics and the patient came to the ED.

**Figure 1:**
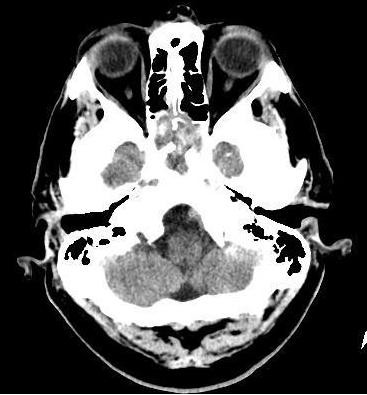
**CT Scan on admission showing opacification of the sphenoid sinuses**.

Vital signs in the ED were temperature of 100.4°F, pulse of 106 bpm, blood pressure 159/64 mmHg, and 18 breaths/min. Physical examination was notable for bilateral tenderness over the maxillary and frontal sinuses. There was no nuchal rigidity and the extra-ocular muscles were intact. The rest of the physical exam was unremarkable. A complete blood count revealed a white blood cell count of 7700 cells/mm^3^; hemoglobin of 15 mg/dL; and platelet count of 182,000/mm^3^. The erythrocyte sedimentation rate (ESR) was 1. The basic metabolic panel was within normal limits. Initial chest x-ray revealed no acute infiltrates. Computed tomography of the head showed complete opacification of the sphenoid sinuses with loss of adjacent walls in the region of the spheno-ethmoidal recesses showing aggressive chronic sinusitis. Computed tomography of the maxillofacial sinuses revealed pansinusitis with complete opacification of bilateral sphenoid sinuses and thickening of the sphenoid sinuses consistent with chronic sinusitis. Magnetic Resonance Imaging (MRI) of the head confirmed the findings of the CT scans. Magnetic Resonance Angiogram (MRA) was also performed which ruled out the presence any aneurysm or stenosis in the CNS vasculature. Magnetic Resonance Venography (MRV) was also performed for the presence of cavernous sinus thrombosis, which was negative. For CT Scan on admission showing opacification of the sphenoid sinuses see Figure 1.

Lumbar puncture was done which showed a clear, colorless CSF with RBC count of 136/mm^3 ^and WBC count of 8/mm^3^. The differential comprised of 80% neutrophils, 10% lymphocytes and 10% monocytes. The CSF chemistry revealed glucose of 70 mg/dl (serum level: 164 mg/dl); protein of 154 mg/dl; and chloride of 125 mg/dl. Gram staining, culture and India ink stain of the CSF were all negative. Based upon these findings, notably the presence of neutrophils in the CSF, a diagnosis of possible bacterial meningitis was made and the patient was started on intravenous vancomycin, ceftriaxone, as well as acyclovir, and was admitted.

While on the medical ward, the patient continued to have persistent headaches. An EEG was conducted and showed no evidence of encephalopathy or epileptiform activity. Interim blood cultures were negative for growth. Fungal blood cultures were also negative. Serum crytptococcal antigen and Lyme antibodies were unremarkable. To further aide with the diagnosis, the otorhinolaryngology service was consulted. Subsequently, he underwent endoscopic drainage of the sphenoid sinuses, which removed a significant amount of yellow, mucopurulent material. The specimen was sent to pathology for analysis. The patient reported a dramatic improvement of the headaches after the procedure and was afebrile. He was discharged home on clindamycin and ciprofloxacin, in addition to his routine medications.

**Figure 2:**
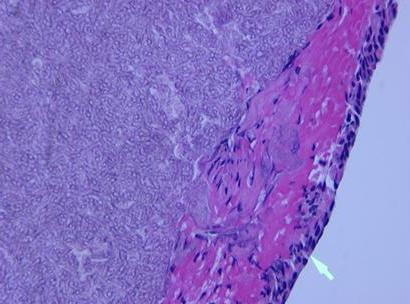
Specimen from sphenoid sinus fixed with H & E stain showing fungal hyphae.

**Figure 3:**
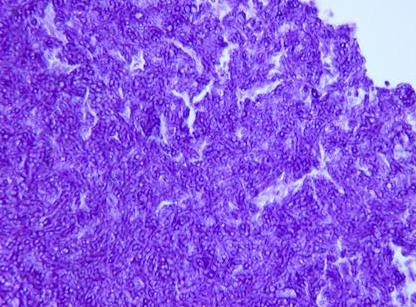
**Specimen from sphenoid sinus fixed with PAS stain demonstrating aspergillus**.

In the interim two days, the pathology report of the sinus specimen was completed and was significant for marked chronic sinusitis with large collections of septate, branching fungi. Fungal stains identified the organisms most consistent with *Aspergillus *species, present focally within the soft tissue of the sinuses. No organisms were specifically seen invading blood vessels. Further fungal cultures for species identification were not carried out. The patient was contacted at home and advised to return to the hospital for treatment of fungal sinusitis. He returned and was started on antifungal therapy with intravenous liposomal amphoterecin B (L-AMB), 500 mg every 24 hours. After a few days of receiving L-AMB, he developed acute renal failure. As a result, L-AMB was switched to intravenous voriconazole 500 mg every 12 hours times 2 doses and then 300 mg every 24 hours. Following this, he began experiencing visual hallucinations, likely due to the antifungal azole. Over the next several days, the patient reported feeling better and subsequently he was discharged on oral voriconazole 200 mg every 12 hours for three weeks. The patient was assessed at follow-up at the end of the three weeks. The patient reported improvement in symptoms gradually over time and a repeat CT scans of the head and sinuses showed clearance of the sinuses. For specimen from sphenoid sinus fixed with H & E stain showing fungal hyphae see Figure 2. See Figure 3 for Specimen from sphenoid sinus fixed with PAS stain demonstrating aspergillus.

Two months later, the patient returned to the ED with severe frontal headache for the previous ten days. He also experienced diminished vision of his left eye. Jaw claudication, photophobia, and nausea were also reported. Further questioning revealed that the patient had gone to another medical institution and was discharged on oral prednisone for presumed temporal arteritis. Blood work was obtained; the patient was started on intravenous dexamethasone and admitted for additional workup. The ESR was 38.

On the medical floor, the patient continued to complain of relentless, worsening headache now associated with bilateral vision diminishment. He was assessed by the neurology and ophthalmology services. The steroid dose was increased. During the following afternoon, the patient was found unresponsive. The patient had no corneal reflexes, no response to noxious stimuli, and fixed, dilated, non-reactive pupils bilaterally. He had no doll's eyes movements. A stroke code protocol was initiated and the patient was intubated for airway protection. Emergent CT scan of the head showed diffuse subarachnoid hemorrhage with a left temporal lobe hematoma and extensive intraventricular hemorrhage. The patient was transferred to the ICU for further management. A repeat CT scan of the head 24 hours later indicated increase in size of the subarachnoid hemorrhage and acute ischemic changes in the distribution of the left middle cerebral artery. The critical condition of the patient did not allow for evaluation with MRI/MRA studies or neurosurgical intervention. Despite aggressive supportive care, the patient expired five days later. An autopsy request was declined by the patient's family.

## Discussion

The development of cerebral hemorrhage signifies a serious complication of invasive fungal infections of the CNS - burst mycotic aneurysms. As the MRA was normal on the first visit to the hospital, and there was no other obvious precipitating factor for the hemorrhage, we strongly suspect the development of a ruptured mycotic aneurysm due to cerebral aspergillosis from direct extension of chronic sphenoid fungal sinusitis, as the most likely cause of the patient's acute demise.

Cerebral angioinvasive aspergillosis is a rare manifestation of disseminated aspergillosis which may result in cerebrovascular accidents in immunocompromised persons, especially those with hematologic malignancies and solid-organ transplants. However, reports of such manifestations in patients with diabetes mellitus are rare [1], thus making our patient an atypical subject. Because the initial chest radiograph was normal in our patient, the fungal dissemination in this patient likely occurred from the paranasal sinuses. This is another atypical facet in this case, as cerebral invasive aspergillosis is usually hematogenously spread from the lungs or GI tract, rather than from direct extension of sinonasal disease [2].

Invasive cerebral aspergillosis is a catastrophic disease, with a high mortality rate of 85%-100%, despite antifungal treatment. It is estimated to occur in 10%-15% of patients with disseminated aspergillosis [3]. Aspergillus species cause an infective vasculopathy leading initially to acute infarction or hemorrhage, and subsequently extending into surrounding tissue as an infectious cerebritis which may evolve into an abscess. Hyphal elements can grow through an artery, thereby damaging the composition of the vessel wall, leading to the development of mycotic aneurysms [4], of the larger arteries, which can rupture and cause massive hemorrhage. The location of the hematoma in this case, in the temporal lobe, goes in favor of mycotic aneurysm rather than that seen usually with berry aneurysms [5].

The clinical diagnosis of cerebral aspergillosis is difficult because the presenting symptoms are nonspecific and fever may be absent. Patient may present with signs and symptoms of sinusitis and in advanced cases, proptosis, cranial nerve palsies, visual deterioration and chemosis may become evident. Cerebral aspergillosis may present with meningitis, cerebritis, infarction, abscess, granuloma, mycotic aneurysms, or solitary mass-like lesion. The most frequent pathologic manifestation is hemorrhagic infarction and abscess [6].

The diagnosis of aspergillosis of the CNS is difficult and is based on a combination of clinical risks, symptoms and signs, culture, histopathology, and detection of fungal components, such as galactomannan or beta-D-glucan [7]. Histological studies done on specimens taken directly from the sinuses may reveal faintly visible characteristic branching septate hyphae at 45° on direct microscopy with H & E stain, but are more readily seen with Gomori's methenamine silver (GMS) stain [8]. Histopathologic studies in acute invasive fungal sinusitis reveal hyphal invasion of the mucosa, submucosa, and blood vessels, including the carotid arteries and cavernous sinuses; vasculitis with thrombosis; hemorrhage; and tissue infarction. The culture of *Aspergillus *spp is becoming increasingly important, given the emergence of antifungal drug-resistant non-fumigatus *Aspergillus *spp, as well as rising frequency of other molds causing invasive disease [9].

Surgery remains the mainstay of treatment in all forms of invasive fungal sinusitis. Aggressive neurosurgical intervention for surgical removal of any abscess, granulomas, focally infracted brain tissue, and correction of underlying risk factors are important. If the infection is known to be due to *Aspergillus *spp, voriconazole should be initiated [10]. The efficacy of voriconazole as primary therapy has been established in clinical trials for invasive aspergillosis [11].

This case report suggests that a mycotic aneurysm was the result of the direct extension of chronic *Aspergillus *sinusitis to the intracerebral circulation in an immunocompetent patient. The implications of this case are noteworthy for several reasons. Chronic sinusitis should be recognized as an early sign of possible aspergillosis and often presents with new-onset, persistent headaches. Our patient had also been taking oral prednisone as an outpatient and was placed on intravenous dexamethasone prior to his deterioration. The steroids may have been a contributing factor to the overall disease progression. In a recent study, Orciuolo et al, showed that the combination of methylprednisolone therapy and local production of gliotoxin (a mycotoxin produced by *Aspergillus fumigatus*) may contribute to increased inflammatory response leading to tissue injury and impaired T-cell immune response, which may be associated with increased morbidity and mortality in cases of invasive aspergillosis [12]. Almost all immunocompetent patients with cerebral aspergillosis in the literature have had the predisposing factors of old age, diabetes, alcoholism, hepatic failure, drug addiction, post-surgical sequelae, or post-traumatic events - many of these risk factors our patient had. This is especially important in diabetic patients which comprise a large portion of our patient population. Also of note was our patient's Egyptian descent. Chronic invasive sinonasal aspergillosis has been documented in immunocompetent patients living in dry-air climates, similar to the weather found in Egypt, such as Sudan, Saudi Arabia, and India [13]. A major reason for the devastating prognosis in cerebral aspergillosis is the delay in the diagnosis. High degree of suspicion of disease is needed, prompt use of imaging and initiation of antifungal therapy are potentially life-preserving tactics because of the difficulty in avoiding a disastrous outcome with invasive cerebral aspergillosis once vascular involvement has occurred.

## Abbreviations

Bpm: beats per minute; °F: degrees Fahrenheit; mmHg: millimeters of mercury; breaths/min: breaths per minute; cells/mm^3^: cells per cubic micrometer; mg/dL: milligrams per deciliter; CT: computed tomography; CNS: central nervous system; CVS: cardiovascular system; GI: gastrointestinal; CSF: cerebrospinal fluid; EEG: electroencephalogram; FDA: Food and Drug Administration; L-AMB: liposomal amphotericin B; ICU: intensive care unit; H & E: hemotoxylin and eosin; PCR: Polymerase Chain reaction; mRNA: messenger ribonucleic acid;

## Consent

Written informed consent was obtained from the patient's next of kin/family for publication of this case report. A copy of the written consent is available for review by the Editor-in-Chief of this journal.

## Competing interests

The authors declare that they have no competing interests.

## Authors' contributions

AK was the major contributor to the Discussion section of the manuscript, conducted literature review, participated in manuscript revisions, and was involved in direct patient care of the patient in the intensive care unit. MP was the major contributor to the Case Presentation section of the manuscript, conducted literature review, and manuscript revisions. NM was involved in direct patient care as the infectious disease consultant and participated in manuscript revisions. All authors have read and approved the final manuscript.
